# Tamoxifen Ameliorates Cholestatic Liver Fibrosis in Mice: Upregulation of TGFβ and IL6 Is a Potential Protective Mechanism

**DOI:** 10.3390/biomedicines10051209

**Published:** 2022-05-23

**Authors:** Dino Šisl, Darja Flegar, Maša Filipović, Petra Turčić, Pavao Planinić, Alan Šućur, Nataša Kovačić, Danka Grčević, Tomislav Kelava

**Affiliations:** 1 Laboratory for Molecular Immunology, Croatian Institute for Brain Research, School of Medicine, University of Zagreb, 10000 Zagreb, Croatia; dino.sisl@mef.hr (D.Š.); darja.flegar@mef.hr (D.F.); masa.filipovic@mef.hr (M.F.); alan.sucur@mef.hr (A.Š.); natasa.kovacic@mef.hr (N.K.); danka.grcevic@mef.hr (D.G.); 2 Department of Physiology, School of Medicine, University of Zagreb, 10000 Zagreb, Croatia; 3 Department of Pharmacology, Faculty of Pharmacy and Biochemistry, University of Zagreb, 10000 Zagreb, Croatia; pturcic@pharma.hr; 4 Department of Physiology, School of Medicine, University of Mostar, 88000 Mostar, Bosnia and Herzegovina; pavaoplaninic98@gmail.com; 5 Department of Anatomy, School of Medicine, University of Zagreb, 10000 Zagreb, Croatia

**Keywords:** liver fibrosis, tamoxifen, DDC model, cholestatic liver disease

## Abstract

The available treatments for cholestatic liver fibrosis are limited, and the disease often progresses to liver cirrhosis. Tamoxifen is a selective modulator of estrogen receptors, commonly used in breast cancer therapy. A recent in vitro study showed that tamoxifen deactivates hepatic stellate cells, suggesting its potential as an antifibrotic therapeutic, but its effects in vivo remain poorly investigated. In the present study, we show that tamoxifen protects against the cholestatic fibrosis induced by a diet supplemented with 0.025% 3,5-diethoxycarbonyl-1,4-dihydrocollidine (DDC). Mice fed with a DDC-supplemented diet for four weeks and treated with tamoxifen developed a significantly milder degree of liver fibrosis than vehicle-treated mice, as evidenced by a lower percentage of Sirius red-stained area (60.4% decrease in stained area in male and 42% decrease in female mice, *p* < 0.001 and *p* < 0.01, respectively) and by lower hydroxyproline content. The finding was further confirmed by qPCR analysis, which showed a lower expression of genes for *Col1a1*, *Acta2*, *Sox9*, *Pdgf*, and *Krt19*, indicating the inhibitory effect on hepatic stellate cells, collagen production, and biliary duct proliferation. The degree of protection was similar in male and female mice. Tamoxifen per se, injected into standard-diet-fed mice, increased the expression of genes for *Il6* (*p* < 0.01 and *p* < 0.001 in male and female mice, respectively) and *Tgfβ* (*p* < 0.01 for both sexes), and had no adverse effects. We showed that tamoxifen sex-independently protects against cholestatic DDC-induced liver fibrosis. The increased expression of Il6 and Tgfβ seems to be a plausible protective mechanism that should be the primary focus of further research.

## 1. Introduction

Primary sclerosing cholangitis and primary biliary cholangitis are two major types of cholestatic liver disease affecting approximately 200–500 individuals per million inhabitants [1]. Currently, available treatments are limited, and the disease often slowly progresses to liver cirrhosis, at which point liver transplantation remains the only available therapy. Unfortunately, the recurrence of the disease occurs in a significant number of transplant recipients. The pathogenesis of cholestatic liver disease is initiated by damage in the small intrahepatic biliary ducts, which is followed by their proliferation and inflammatory response, leading to the activation of hepatic stellate cells. The activated hepatic stellate cells start expressing alpha smooth muscle actin (αSMA), proliferate, and differentiate into collagen-secreting myofibroblasts [2,3].

The most common animal model used to study cholestatic disease is a diet containing 3,5-diethoxycarbonyl-1,4-dihydrocollidine (DDC), which induces hepatobiliary injury and closely mimics key events in the development of biliary fibrosis [4,5]. Exposure to a DDC-supplemented diet causes progressive accumulation of protoporphyrin, which is excreted by bile, leading to the obstruction of the lumen of the smaller branches of the biliary tree, which is followed by their proliferation, inflammatory response, and the development of hepatic fibrosis [6].

Tamoxifen is a selective modulator of estrogen receptors, commonly used in breast cancer therapy. It acts by competing with estradiol for the binding site [7]. A recent in vitro study showed that tamoxifen mechanically deactivates hepatic stellate cells, suggesting its potential as an antifibrotic therapeutic [8]. However, the antifibrotic effects of tamoxifen in vivo remain poorly investigated. Kulcsar et al. reported that tamoxifen has a protective role in a rat model of carbon tetrachloride-induced fibrosis [9], but in a later research, Xu et al. found the opposite effect [10]. Studies on murine models have suggested that tamoxifen might have a hepatoprotective role in lipopolysaccharide (LPS)-induced acute liver failure, steatosis, and non-alcoholic steatohepatitis [11,12].

In addition to its use in a clinical setting, tamoxifen is also used in basic biomedical research, where it is applied in transgenic mouse strains that use the Cre/lox system to activate or inhibit selected gene targets in an inducible manner [13]. The inducible modulation of gene expression following the application of tamoxifen has led to many breakthrough scientific discoveries, but constant caution is necessary, as tamoxifen per se is not an inert substance and can cause confounding effects. Studies of liver fibrosis performed in such transgenic animals require the administration of multiple tamoxifen injections [14], which imposes the need to clearly define the effects of tamoxifen on fibrogenic processes in order to avoid biased experimental design. Of special importance are the findings of previous investigations that have established the modulatory role of tamoxifen on the expression of transforming growth factor β (TGFβ) [15,16]. This cytokine is involved in fibrogenic hepatic processes, and the findings of recent studies suggest that it might have an inhibitory effect on bile duct proliferation, which is of critical importance for the development of DDC-induced cholestatic fibrosis [17,18].

In the present study, we demonstrate that tamoxifen treatment ameliorates DDC-induced liver fibrosis, and we analyze its modulatory effect on the initial damage of biliary ducts and subsequent fibrogenic pathways. As tamoxifen effects are often sex-dependent [15], we have conducted experiments on both female and male animals to define possible sexual dimorphism.

## 2. Materials and Methods

### 2.1. Mice

All the animal experiments in this study were approved by the National Ethics Committee. We adhered to all the relevant guidelines and regulations for the use of laboratory animals (EU Directive 2010/63/EU for animal experiments, the National Institutes of Health Guide for the Care and Use of Laboratory animals) and ARRIVE (Animal Research: Reporting In Vivo Experiments) guidelines for reporting animal research. Male and female 6- to 8-week-old C57Bl/6 mice were used in these experiments. The mice were bred and housed in the animal facility of the Croatian Institute for Brain Research, School of Medicine, University of Zagreb (Zagreb, Croatia) under standard conditions.

### 2.2. DDC-Food Supplementation

Standard food pellets (4RF21) and same pellets supplemented with 0.1% and with 0.025% DDC (Sigma-Aldrich, Burlington, MA, USA, cat. No.: 137030) were obtained from Mucedola (Milan, Italy).

### 2.3. Fibrosis Induction and Experimental Design

The mice were divided into four groups. The effect of tamoxifen on fibrosis development was investigated in two DDC-fed groups. These groups (*N* = 7–13 animals per group) were fed with 0.025% DDC-supplemented pellets (0.1% DDC-supplemented pellets were used in the initial experiment, see below) for four weeks and were given either tamoxifen (Sigma-Aldrich, Burlington, MA, USA, cat. no. T5648) (75 µg/g) or vehicle (corn oil, Sigma-Aldrich, Burlington, MA, USA, cat. No. C8267) intraperitoneally per day, starting one day after introducing DDC feeding and ending one day before sacrifice. The effect of tamoxifen per se was studied in two standard diet-fed groups that were given either tamoxifen or vehicle (*N* = 4 animals per group) in the same manner as in the DDC-fed groups) [19,20]. At the experimental endpoint, the mice were sacrificed, and blood (for determination of aminotransferase activities in sera) and liver tissue samples (for qPCR analysis, histology, immunohistochemistry, and hydroxyproline content determination) were harvested and stored until analysis. In accordance with the institutional standard of care and ethical policy, the health of the animals was monitored each day, and animals that, due to health deterioration, reached the criteria for a humane endpoint were immediately excluded from the experiment and euthanized. The euthanasia was necessary only in mice fed 0.1% DDC supplementation.

### 2.4. Determination of Hydroxyproline Content in Liver Tissue

The hydroxyproline content in the liver tissue was determined using a commercial kit according to the manufacturer’s instruction (Hydroxyproline Assay Kit, cat. No. MAK008, Sigma-Aldrich, Burlington, MA, USA) with slight modification [21]. Briefly, livers were snap-frozen in liquid nitrogen and stored at −80 °C until the analysis. After being weighed, the livers were homogenized in ultrapure water (10 mg of tissue per 100 µL of water). Following homogenization, an equal volume of 12 M HCL was added, and samples were transferred to a pressure-tight polypropylene vial and hydrolyzed at 100 °C for 20 h. After cooling the samples and centrifugation (10,000× *g* for 3 min, room temperature), the supernatant was transferred to a new tube and completely dried under a vacuum at 60 °C. After that, the samples were incubated in 100 µL of Chloramine T/Oxidation Buffer Mixture at room temperature for 5 min, and then we added 100 µL of freshly diluted 4-(dimethylamino) benzaldehyde (DMAB) reagent. After incubation (90 min at 60 °C), the absorbance was read at 560 nm, and the concentration was determined using a standard curve obtained by the measurement of hydroxyproline standards provided in the kit.

### 2.5. Histology—Sirius Red Staining

The liver tissue samples were fixed in 4% paraformaldehyde overnight and dehydrated in increasing ethanol concentrations (70%, 96%, and 100%), transferred to benzene for 30 min, and embedded in paraffin overnight. Sections were cut (5 µm) with a rotational microtome (Leica SM 2000 R, Leica Biosystems, Nussloch, Germany), and finally stained with Sirius red dye. Two blinded researchers (D.Š. and T.K.) independently analyzed the slides under a light microscope (Axiovert 200; Carl Zeiss, Oberkochen, Germany) equipped with a camera. Photographs were taken, and the red-stained surface area was quantified using the Image J processing program (ImageJ 1.52a) [22].

### 2.6. Immunohistochemistry

The liver sections were incubated overnight with primary rabbit anti-αSMA (D4K9N, Cell Signaling #19245, Danvers, MA, USA) or rabbit anti-keratin19 (D4G2, Cell Signaling #12434) antibody, which is specific for both keratin 17 and keratin 19, but in the liver, keratin 19 is predominant [23]. After washing, the slides were incubated with HRP (horseradish peroxidase) conjugated secondary anti-rabbit antibody (MACH 1 Universal HRP-Polymer, ref no. MRH538L10, Biocare Medical, Pacheco, CA, USA) and stained with diaminobenzidine (DAB). The slides were then analyzed under a light microscope equipped with a camera (Axiovert 200; Carl Zeiss, Oberkochen, Germany) [24].

### 2.7. Determination of Serum Activity of Aminotransferases

The serum was separated by centrifugation after clot formation and stored at −20 °C until analysis. The alanine-aminotransferase (ALT) and aspartate-aminotransferase (AST) serum levels were determined by standard laboratory techniques in a clinical diagnostic laboratory using an Olympus AU400 analyzer [24].

### 2.8. Quantitative PCR Gene Expression Analysis

For quantitative PCR (qPCR), total RNA was isolated from the liver tissue samples using TRI reagent (cat. no. T9424, Sigma-Aldrich, Burlington, MA, USA) and quantified on a Nanodrop spectrophotometer (Thermo Fisher Scientific, Waltham, MA, USA). A High-Capacity RNA-to-cDNA Kit (Applied Biosystems) was used for reverse transcription and generation of cDNA. The cDNA was amplified by ABI Prism 7500 system (Applied Biosystems, Waltham, MA, USA) using TaqMan Gene Expression Master Mix (cat. no. 4369514, Applied Biosystems, Waltham, MA, USA) and commercially available TaqMan^TM^ Gene Expression Assays (Applied Biosystems, Waltham, MA, USA). The analyzed genes included *Col1a1* (Assay ID: Mm00801666_g1), *Acta2* (Assay ID: Mm01546133_m1), *Pdgfb* (Assay ID: Mm00440677_m1), *Tgfβ1* (Assay ID: Mm01178820_m1), *Il6* (Assay ID: Mm00446190_m1), *Sox9* (Assay ID: Mm00448840_m1), *Tnfα* (Assay ID: Mm00443258_m1), *Hes1* (Assay ID: Mm01342805_m1), *Hey1* (Assay ID: Mm00468865_m1), and *Krt19* (Assay ID: Mm00492980). Gene expression was calculated using the ∆∆CT method and normalized to the expression level of the housekeeping gene (*GAPDH*) using the standard-food-vehicle-treated group as a reference [24].

### 2.9. Statistical Analysis

Data are presented as mean with SD. The normality of distribution was tested by the Shapiro–Wilk test, and the difference between the groups was tested by Student’s *t*-test. Variables with non-normal distribution (due to one outlier value greater than mean + 3SD) were log-transformed prior to the Student’s *t*-test calculation to normalize the distribution. GraphPad Prism version 6 for Windows (GraphPad Software Inc., La Jolla, CA, USA) software was used for analysis, and a *p*-value <0.05 was considered statistically significant.

## 3. Results

### 3.1. DDC-Supplementation Model—Dose Adjustment

The initial experiment was performed with 0.1% DDC-supplemented pellets, which is the most common dose described in the literature [4,5]. However, under this treatment schedule, more than 50% of the mice needed to be sacrificed within the first 10 days for ethical reasons because of reaching the criteria for the humane endpoint. We ordered new pellets from the supplier, supplemented with 0.025% DDC, and in the repeated experiment, the new regiment allowed all animals to survive until the planned endpoint of the experiment (4 weeks) without significant health deterioration. The DDC-fed mice developed cholestatic liver fibrosis with all pathohistological, biochemical, and genetic findings closely resembling those described in the literature. We assumed that a reduction in the DDC content was necessary because DDC supplementation in industrially produced pellets is more effective than manual laboratory supplementation described in the literature.

### 3.2. Tamoxifen Treatment Ameliorates Liver Fibrosis Development

**Figure 1:**
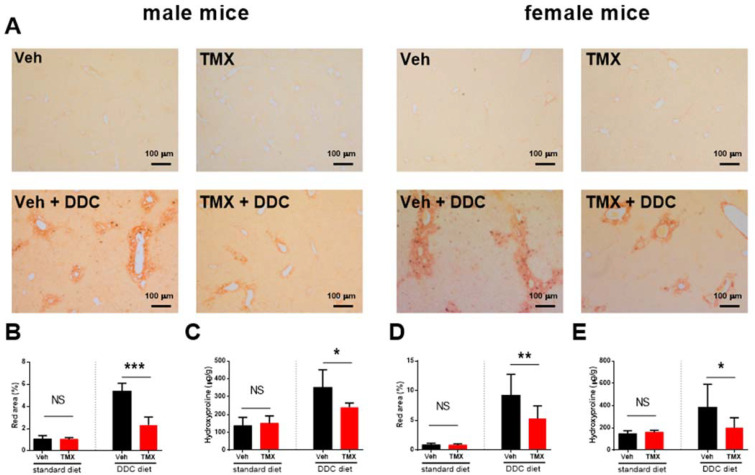
Tamoxifen ameliorates DDC-induced fibrosis. Male or female mice were fed standard or DDC-supplemented diet as indicated, and treated with vehicle (black bars) or tamoxifen (red bars). Light microscope photograph of representative Sirius red-stained liver section for each group is shown (**A**). Red area was quantified using Image J software and hydroxyproline content was determined in liver tissue using the commercially available kit (**B**–**E**). Data represent mean + SD, Student’s *t*-test was used for comparison between the groups, * *p* < 0.05, ** *p* < 0.01, *** *p* < 0.001, NS—nonsignificant, TMX—tamoxifen, DDC—3,5-diethoxycarbonyl-1,4-dihydrocollidine.

**Figure 2:**
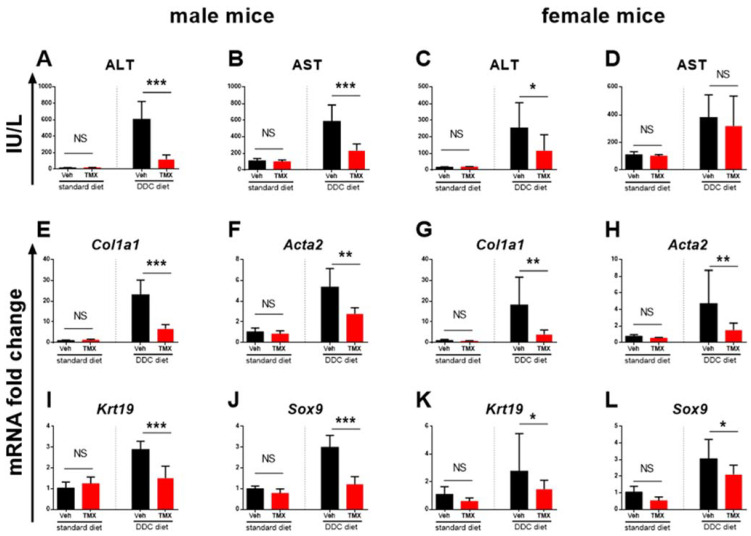
Tamoxifen treatment decreases aminotransferase activity in plasma and expression of genes involved in pathogenesis of biliary fibrosis in liver tissue. Male or female mice were fed standard or DDC-supplemented diet as indicated, and treated with vehicle (black bars) or tamoxifen (red bars). Aminotransferase activity was determined in sera (**A**–**D**). Gene expression was determined in liver tissue using qPCR (**E**–**L**). Data represent mean + SD, Student’s *t*-test was used for comparison between the groups, * *p* < 0.05, ** *p* < 0.01, *** *p* < 0.001, NS—nonsignificant, ALT—alanine aminotransferase, AST—aspartate aminotransferase, *Col1a1*—collagen 1a1, *Acta2*—actin alpha 2, smooth muscle, *Sox9* —SRY (sex-determining region Y)-box 9, *Krt19*—keratin 19, TMX—tamoxifen, DDC—3,5-diethoxycarbonyl-1,4-dihydrocollidine.

**Figure 3:**
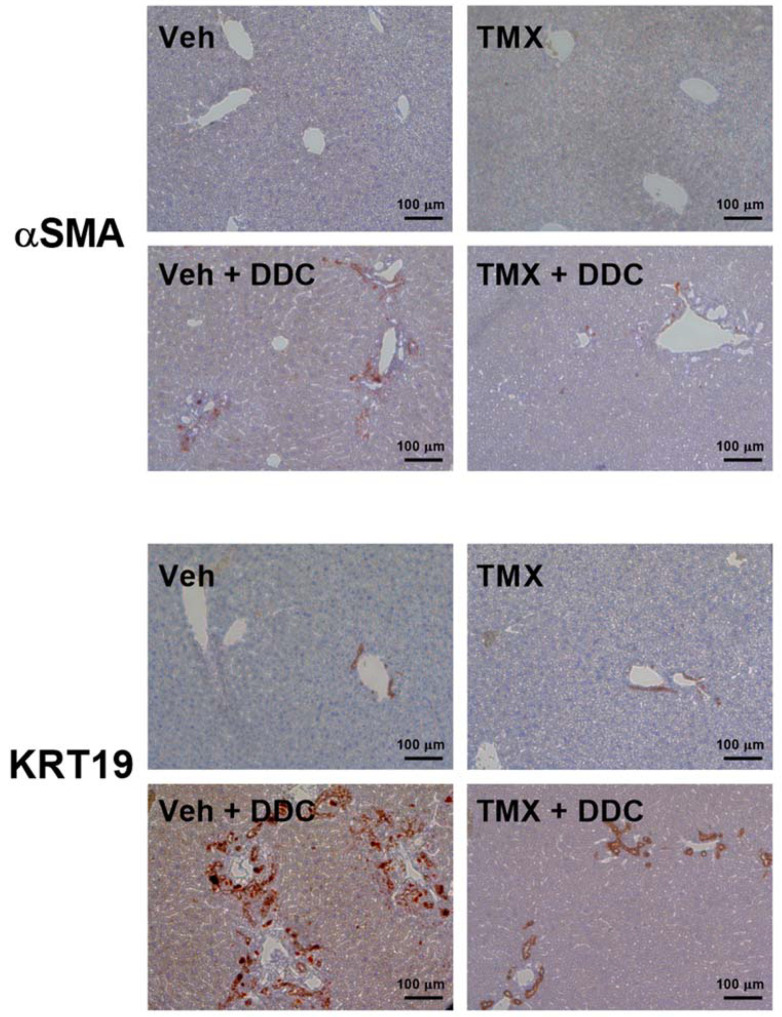
Tamoxifen treatment decreases protein expression of αSMA and KRT19. Mice were fed standard or DDC-supplemented diet, and treated with tamoxifen or vehicle, as indicated. Liver sections were first incubated with anti-αSMA or anti-KRT19 antibody as indicated, and then incubated with HRP-conjugated secondary antibody and stained with diaminobenzidine (DAB). Standard diet-fed mice had low expression of αSMA and KRT19 regardless of treatment. DDC-feeding increased expression of αSMA and KRT19 in vehicle-treated mice, as shown by brown staining and tamoxifen treatment significantly reduced this increase. Sections are from male mice, and the same pattern was obtained in female mice (not shown). The anti-keratin antibody stains both keratin 17 and keratin 19, but in liver, keratin 19 is predominant. αSMA—alpha smooth muscle actin, Krt19—keratin 19, TMX—tamoxifen, DDC—3,5-diethoxycarbonyl-1,4-dihydrocollidine.

**Figure 4:**
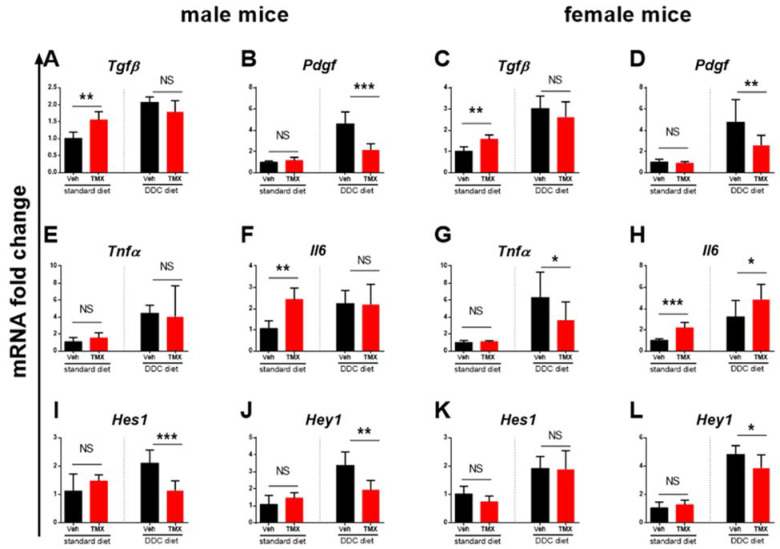
Expression of inflammatory and Notch-pathway related genes. Male or female mice were fed standard or DDC-supplemented diet as indicated, and treated with vehicle (black bars) or tamoxifen (red bars). Gene expression of Tgfβ, Pdgf (**A**–**D**), Tnfα, Il6 (**E**–**H**), *Hes1,* and *Hey1* (**I**–**L**) was determined in liver tissue using qPCR. Data represent mean + SD, Student’s *t*-test was used for comparison between the groups, * *p* < 0.05, ** *p* < 0.01, *** *p* < 0.001, NS—nonsignificant. Tgfβ—transforming growth factor β, Pdgf—platelet-derived growth factor, Tnfα—tumor necrosis factor alpha, Il6—interleukin 6, Hes1—hes family bhlh transcription factor 1, Hey1—Hairy/enhancer-of-split related with YRPW motif protein 1, TMX—tamoxifen, DDC—3,5-diethoxycarbonyl-1,4-dihydrocollidine.

The DDC-fed mice (0.025% DDC supplementation) treated with tamoxifen developed a significantly milder degree of liver fibrosis than vehicle-treated DDC-fed mice, as evidenced pathohistologically by a lower percentage of Sirius red-stained area (*p* < 0.001 and *p* < 0.01 for male and female mice, respectively) and biochemically by lower hydroxyproline content in the liver tissue (*p* < 0.05 for male and for female mice, Figure 1). The finding was further confirmed by qPCR analysis, which showed lower gene expression of *Col1a1*, *Acta2*, and *Sox9*, indicating the inhibitory effect on hepatic stellate cell activity and collagen production. Tamoxifen also reduced the expression of *Krt19*, a characteristic marker of biliary duct proliferation. These findings are further supported by lower aminotransferase activities in the sera of tamoxifen-treated DDC-fed animals (Figure 2). The immunohistochemical analysis of liver sections confirmed lower protein expression of both αSMA and KRT19 (Figure 3). Furthermore, tamoxifen treatment also prevented the DDC-induced increase in the expression of the *Pdgf* gene and the Notch signaling pathway-related genes *Hes1* and *Hey1*. The latter effect was more pronounced in male mice. The effect of tamoxifen on DDC-induced expression of pro-inflammatory genes for *Tnfα* (inhibition) and *Il6* (stimulation) was significant only in female mice (Figure 4).

### 3.3. Tamoxifen Increases Expression of Il6 and Tgfβ in the Livers of Standard Diet-Fed Mice

The analysis of two standard diet-fed groups (without DDC) found no difference between the tamoxifen- and vehicle-treated mice in liver histology (Figure 1), in liver hydroxyproline content or serum aminotransferase activity, and in *Col1a1*, *Acta2*, *Krt19*, and *Sox9* gene expression (Figure 2), suggesting no toxic or profibrotic effect of tamoxifen applied per se. This conclusion was further confirmed by the pathohistology of the liver tissue, as no difference in collagen, Krt19, and αSMA expression was found between the two standard diet-fed groups (Figure 1 and Figure 3). On the other hand, treatment with tamoxifen significantly increased the expression of *Il6* and *Tgfβ* in the livers of both male and female standard diet-fed mice (*p* < 0.01 for both genes in any sex). No influence of tamoxifen alone (without DDC) on the expression of genes associated with Notch signaling pathway *Hes*1 and *Hey1* or on *Pdgf* and *Tnfα* gene expression was found (Figure 4, *p* > 0.05 in both male and female mice).

## 4. Discussion

In the present study, we further explore the antifibrotic effect of tamoxifen, which was recently suggested by Cortes et al. in in vitro experiments [8]. We show, for the first time, that tamoxifen has a protective effect in a DDC-induced model of cholestatic liver injury, and that the ameliorative effect is of a similar degree in both male and female mice.

All major fibrotic indices in DDC-fed animals (histological, genetic, and biochemical) were milder in the group that received tamoxifen than in the vehicle-treated group. Tamoxifen given alone (with a standard diet) did not have any influence on liver histology, hydroxyproline content, or serum aminotransferase levels, suggesting no toxic or profibrotic effect. It caused, however, an increase in *Tgfβ* and *Il6* expression, whereas the expression of all other investigated genes remained similar to the vehicle-treated group.

The increased expression of *Tgfβ* following the tamoxifen treatment of standard diet-fed animals is in accordance with previous reports about the effects of tamoxifen in various other organs, such as the mammary gland, lungs, and aorta [15,16,25]. As TGFβ is a well-known contributor to the activation of hepatic stellate cells and fibrosis development [26], this effect seems contradictory. However, the protective effect of TGFβ has recently been reported in a bile duct ligation model [17]. Furthermore, it was described that specific TGFβ loss in epithelial cells does not contribute to fibrosis but does protect mice from cholangiocarcinoma through an inhibitory effect on cholangiocyte proliferation in the DDC animal model [18]. As the proliferation of cholangiocytes is important for the pathogenesis of DDC-induced fibrosis, the protective effect of tamoxifen might be mediated through *Tgfβ* expression.

**Figure 5:**
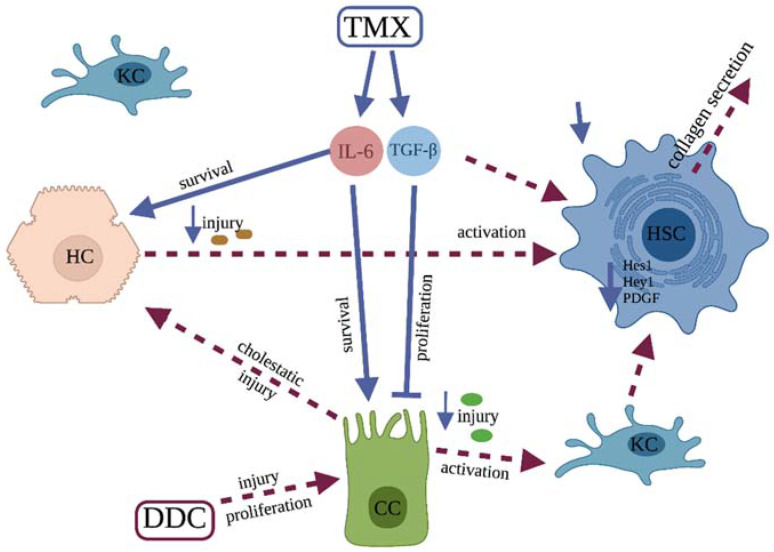
Proposed mechanism of the protective effect of tamoxifen. Tamoxifen treatment leads to Increased expression of TGF-β which is one of the activation signals for hepatic stellate cells, but this action is overridden through inhibitory effect on cholangiocyte proliferation. Furthermore, increased expression of IL-6 promotes survival of both hepatocytes and cholangiocytes thereby decreasing the profibrotic activation of Kupffer and hepatic stellate cells. Tgfβ—transforming growth factor β, Pdgf—platelet-derived growth factor, Il6—interleukin 6, Hes1—hes family bhlh transcription factor 1, Hey1—Hairy/enhancer-of-split related with YRPW motif protein 1, TMX—tamoxifen, DDC—3,5-diethoxycarbonyl-1,4-dihydrocollidine; KC—Kupffer cel; HSC—hepatic stellate cell; HC—hepatocyte; CC—cholangiocyte. Created with BioRender.com, accessed on 24 April 2022.

Similar to *Tgfβ*, the expression of *Il6* was also increased in tamoxifen-treated standard diet-fed animals. The profibrogenic role of IL-6 has been described in the liver and other organs [27], but its effect on cholestatic liver fibrosis seems to be protective. Mair et al., reported that inhibition of the IL-6 signaling pathway by the conditional inactivation of Stat3 in hepatocytes and cholangiocytes strongly aggravates fibrosis in the mdr2^−/−^ transgenic fibrosis model [28]. A similar effect was shown by Plum et al. in the DDC model [29]. It is important to notice that, in our experiments, there was no significant difference in the expression of *Tgfβ* and *Il6* between the vehicle- and tamoxifen-treated DDC-fed animals, as a similar increase in expression occurred in both groups. We assume that in the vehicle-treated group, the increased expression occurred due to DDC, but in the tamoxifen-treated group, the increase was also mediated by tamoxifen, since the increase was larger than expected for the degree of fibrosis. Altogether, we may hypothesize that the protective effect of tamoxifen might be mediated through a stimulatory effect on *Il6* and *Tgfβ* secretion, shown in standard diet-fed mice, suggesting these pathways as a primary focus for further research (Figure 5).

The upregulation of PDGF is a characteristic finding in experimental liver fibrosis models as well as in human liver fibrotic samples. PDGF exerts a potent proliferative effect on hepatic stellate cells, leading to increased extracellular matrix production. Inhibitors of the PDGF pathway show a promising effect in in vitro experiments, but their use in vivo is still hampered by the lack of efficiency as well as by unwanted side effects [30,31]. As expected, DDC feeding upregulated PDGF expression in our experiments, and we also found a lower expression of PDGF in tamoxifen-treated DDC-fed animals. However, tamoxifen per se, applied to standard diet-fed animals, had no influence on *Pdgf* expression. Therefore, we can exclude the possibility that tamoxifen acts through a direct blockade of PDGF expression. The lower expression of PDGF found in tamoxifen-treated DDC-fed mice is most probably a secondary consequence of lower cholangiocyte proliferation mediated by the previously described effects of IL-6 and TGF-β, but the possibility that tamoxifen acted protectively by interfering with pathways that mediate DDC-induced overexpression of PDGF cannot be excluded completely.

Investigations conducted in the last decade have established the activation of the Notch signaling pathway as an important contributor to liver fibrosis initiation and progression. The increased expression of Notch-related genes *Hes* and *Hey* occurs in various liver fibrosis experimental models and in human fibrotic samples [32,33,34]. Patients suffering from Alagille syndrome, caused by a mutation in the gene for the Notch signaling pathway, are less sensitive to hepatic fibrosis development [35]. Novel findings also suggest Notch involvement in the progression of liver cirrhosis and development of hepatocellular carcinoma [36,37]. The inhibitors of gamma secretase, a key enzyme in Notch signal transduction, were reported to ameliorate liver fibrosis in animal models, but the lack of specificity still prevents the use of such therapy in humans [38]. Our experiments showed that the Notch signaling pathway is induced in the DDC model, as evidenced by an increase in the primary downstream genes *Hes1* and *Hey*1, and that tamoxifen decreases this activity when applied to DDC-fed mice. The effect was more visible in male mice. However, tamoxifen per se, applied to standard diet-fed animals, had no inhibitory effect on the expression of Notch genes, suggesting no direct interaction with Notch pathway activity. Similar to PDGF, the lower activity of the Notch signaling pathway found in tamoxifen-treated DDC-fed mice is most probably a secondary consequence of lower cholangiocyte proliferation, mediated by the effects of IL-6 and TGF-β, but the possibility of indirect interference should not be completely excluded. To the best of our knowledge, there are no published studies so far that reported interaction between the tamoxifen and Notch signaling pathway.

The findings of previous studies indicate that tamoxifen can often induce different effects in male and female animals. For example, a sex-dependent effect was recently reported in the glucose tolerance model and in the unilateral ureteral obstruction model of kidney fibrosis [39,40]. However, we found a similar degree of antifibrotic effect in both male and female mice, suggesting no major sex dependency. Nevertheless, there were minor discrepancies between male and female mice in the expression of genes associated with fibrogenic mechanisms. Particularly, the expression of genes for Notch-related *Hes1* and *Hey1* decreased more in male mice, while the expression of *Tnfα* was decreased only in female mice.

In basic medical research, tamoxifen is extensively used to induce the Cre recombinase system, enabling the researcher to activate or inhibit selected molecular targets at a specific time point, specific tissue, or cell line. In the field of liver fibrosis, a particularly large number of tamoxifen injections are used to ensure the Cre recombinase induction in activated hepatic stellate cells [14,41]. Our results indicate that there are possible confounding interactions of tamoxifen, especially on IL-6- and TGF-β-mediated effects, emphasizing a need for caution in experimental design and result interpretation. In the present investigation, we used the lower dose of tamoxifen commonly used in Cre induction experiments, finding no gross adverse effects. The toxicity and effectiveness of tamoxifen are variously reported in the literature and are strain-dependent. Some researchers reported no toxicity when this or higher doses were applied to B6 wild-type mice [19,42], while in some transgenic mice, serious toxicity occurred even with lower doses [43]. Therefore, to define the further translational potential of tamoxifen treatment, it is also necessary to assess the effectiveness of various doses and application routes.

## 5. Conclusions

We showed that tamoxifen sex-independently protects against cholestatic DDC-induced liver fibrosis. We have not defined the precise mode of action, but the increased expression of *Il6* and *Tgfβ* seems to be a plausible protective mechanism that should be the primary focus of further research. The proposed mechanism of action is shown schematically in Figure 5. The possibility of protection through an inhibitory effect on DDC-induced expression of *Pdgf* and Notch-related genes should also be considered.

## Data Availability

Data are contained within the article. Any additional data are available from the corresponding author upon reasonable request.
